# The complete chloroplast genome of *Acanthopanax brachypus* (Araliaceae), a famous medicinal plant in China

**DOI:** 10.1080/23802359.2020.1787888

**Published:** 2020-07-09

**Authors:** Xiao-Bin Ou, Dan-Hua Zhang

**Affiliations:** a China College of Life Sciences & Technology, Longdong University, Gansu, China; b College of Life Sciences, Zhejiang University, Hangzhou, China

**Keywords:** *Acanthopanax brachypus*, chloroplast genome, Araliaceae

## Abstract

*Acanthopanax brachypus* (Araliaceae) is an important medicinal plant originated from China. Here, we reported the first chloroplast genome sequence of *A. brachypus*. The plastome of *A. brachypu* is 156,802 bp in length, including a large single-copy (LSC) of 86,742 bp, a small single-copy (SSC) of 18,184 bp, and a pair of inverted repeat regions (IRa and IRb) of 25,938 bp. It contains 113 unique genes consisting of 80 protein-coding genes, 29 tRNA genes, and 4 rRNA genes.

*Acanthopanax brachypus* (Araliaceae) is a small perennial deciduous shrub and its distribution is extremely narrow in the world, mainly distributed in Shaanxi, Gansu, Ningxia and other places on the Loess Plateau in Northwest China and it is a unique folk medicinal plant in China. Its medicinal parts are mainly stem skin and root, which have the functions of Invigorating Qi and spleen, nourishing heart and mind, relieving depression and blood. According to the research, the extract of its rhizome not only has the treatment of neurasthenia, male sexual dysfunction, secondary hypertension, hypotension, leukopenia and other diseases, but also has the anti-cancer and anti-cancer effect; the alcohol extract of its skin has the anti-inflammatory effect, and can also inhibit adjuvant arthritis. Here, we reported the first chloroplast genome of *A. brachypus*, and reconstructed the phylogenetic relationship with other Araliaceae species. The chloroplast genome sequence of *A. brachypus* would provide abundant genomic resources for utilization and breeding of this species.

The leaves of *A. brachypus* was collected from Qingyang, Gansu, China(N36°05′45.2″ E108°21′2.4″). The voucher specimen was deposited in the Herbarium of Longdong University (Accession NO. XB20200318), Qingyang, Gansu, China. The leaves were dried with silica gel and Genomic DNA was extracted using a standard CTAB method (Murray and Thompson 1980). Genome sequencing was conducted on HiSeq^TM^2500 (Illumina, San Diego, CA, USA) with 150 bp paired-end sequencing. The complete plastome sequence was constructed using GetOrganelle (Jin et al. 2018) and annotated using Geneious Prime 2019.1.1 (www.geneious.com) and the new annotated plastome sequence was deposited in the GenBank (MN527993).

The complete chloroplast genome of *A. brachypus* was 156,802 bp long. The genome consists of a pair of inverted repeat regions (IR) of 25,938 bp separated by a large single-copy (LSC) of 86,742 bp and a small single-copy (SSC) of 18,184 bp. *Acanthopanax brachypus* encoded a total of 113 genes and there were 80 protein-coding genes (CDS), 29 transfer RNA (tRNA) genes, and 4 ribosomal RNA (rRNA) genes.

In order to identify the systematic position of *A. brachypus,* we conducted a phylogenetic analysis using whole chloroplast genomes of *A. brachypus* and the other species. The sequences were aligned using MAFFT 7.017 (Nakamura et al. 2018). The best-fitting model of nucleotide substitution was GTR + G, as determined by the Akaike Information Criterion (AIC) in jModelTest v. 2.1.7 (Darriba et al. 2012). ML (maximum likelihood) analysis was conducted using RAxML-HPC v. 8.2.8 with 1000 bootstrap replicates on the CIPRES Science Gateway website (Miller et al. 2010). Phylogenetic result strongly supported *A. brachypus* belongs to the family Araliaceae (Figure 1), which is consistent with the previous studies based on combined chloroplast genes of Asteraceae (Panero & Funk 2008).

**Figure 1.**
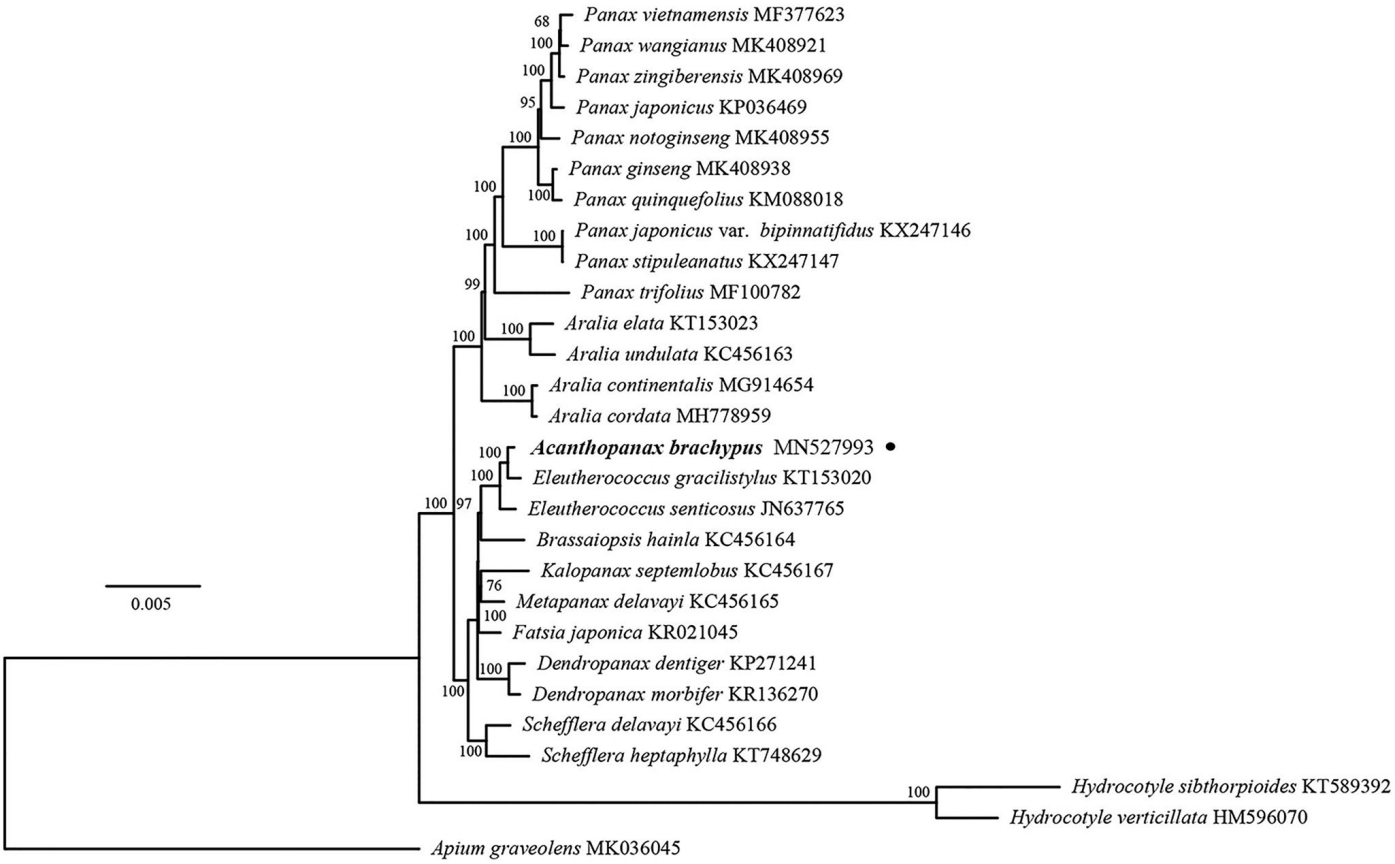
Phylogenetic tree using maximum likelihood (ML) based on plastomes with 1000 bootstrap replicates. Relative branch lengths are indicated. Numbers near the nodes represent ML bootstrap values.

## Data Availability

The authors confirm that the data supporting the findings of this study are available within the article (https://www.ncbi.nlm.nih.gov/nuccore/MN527993.1/).
